# Unbalanced production of LTB_4_/PGE_2_ driven by diabetes increases susceptibility to cutaneous leishmaniasis

**DOI:** 10.1080/22221751.2020.1773744

**Published:** 2020-06-11

**Authors:** Icaro Bonyek-Silva, Sara Nunes, Reinan L. Santos, Filipe R. Lima, Alexsandro Lago, Juliana Silva, Lucas P. Carvalho, Sergio M. Arruda, Henrique C. Serezani, Edgar M. Carvalho, Claudia I. Brodskyn, Natalia M. Tavares

**Affiliations:** a Gonçalo Moniz Institute, Oswaldo Cruz Foundation (FIOCRUZ), Salvador, Brazil; b Federal University of Bahia (UFBA), Salvador, Brazil; c Division of Infectious Diseases, Department of Medicine, Vanderbilt University Medical Center, Nashville, TN, USA; d National Institute of Science and Technology (INCT) in Tropical Diseases, Salvador, Brazil; e National Institute of Science and Technology (INCT), Institute of Investigation in Immunology (iii), São Paulo, Brazil

**Keywords:** Diabetes, human leishmaniasis, *Leishmania braziliensis*, lipid mediators, LTB_4_, PGE_2_

## Abstract

Poorly controlled diabetes mellitus leads to several comorbidities, including susceptibility to infections. Hyperglycemia increases phagocyte responsiveness, however immune cells from people with diabetes show inadequate antimicrobial functions. We and others have shown that aberrant production of leukotriene B_4_ (LTB_4_) is detrimental to host defense in models of bacterial infection. Here, we will unveil the consequences of high glucose in the outcome of *Leishmania braziliensis* skin infection in people with diabetes and determine the role of LTB_4_ in human phagocytes. We show that diabetes leads to higher systemic levels of LTB_4_, IL-6 and TNF-α in cutaneous leishmaniasis. Only LTB_4_ correlated with blood glucose levels and healing time in diabetes comorbidity. Skin lesions of people with leishmaniasis and diabetes exhibit increased neutrophil and amastigote numbers. Monocyte-derived macrophages from these individuals showed higher *L. braziliensis* loads, reduced production of Reactive Oxygen Species and unbalanced LTB_4_/PGE_2_ ratio. Our data reveal a systemic inflammation driven by diabetes comorbidity in opposition to a local reduced capacity to resolve *L. braziliensis* infection and a worse disease outcome.

## Introduction

Diabetes mellitus is a metabolic disorder characterized by hyperglycemia, which occurs when insulin is not produced (Type 1) or inefficiently recognized (Type 2) [1,2]. Both types of diabetes are associated with secondary complications, such as cardiovascular diseases, retinopathy, nephropathy, neuropathy, reduced wound healing and increased susceptibility to infections, particularly in the skin [1–7]. The recent increase in the burden of diabetes [8,9] and reports of abnormal cases of cutaneous leishmaniasis (CL) in individuals with diabetes [10,11] led us to investigate the consequences of association of these diseases. The main goal of this study is to evaluate the influence of diabetes and its inflammatory mediators in the outcome of *Leishmania braziliensis* skin infection.

Hyperglycemia is thought to cause a state called sterile inflammation, characterized by a low-grade inflammatory response [12]. Although inflammation is crucial to clear pathogens and to induce tissue repair, chronic and sustained inflammatory responses cause tissue damage, leading to immunopathology and worse disease outcome. The tissue damage results from increased production of TNF-α, Interleukin-1β, metalloproteases and recently, we have shown that lipid mediators, such as leukotrienes (LTB_4_), when produced in abundance, increases susceptibility to skin infection in experimental models of diabetes [13–15]. These inflammatory mediators are also involved in CL caused by *Leishmania braziliensis,* the causative agent of CL in Brazil, resulting in skin ulcerative lesions [16]. Histological analysis of CL ulcers revealed rare presence of parasites and an intense inflammatory infiltrate, which can lead to a delayed healing process and chronic lesions [17]. In this context, we and others also have shown the participation of lipid inflammatory mediators, including LTB_4_ and Prostaglandin E_2_ (PGE_2_) in phagocyte antimicrobial effector functions upon infection of different pathogens, including *Leishmania* spp*.* [18–21].

Lipid mediators are produced from the metabolism of arachidonic acid (AA) present in the cell membrane in response to several stimuli, such as antigens, microbes, cytokines and osmotic challenge [22]. After AA metabolism, leukotrienes (LTs) and prostaglandins (PGs) are produced from lipoxygenase (LO) or cyclooxygenase (COX), respectively [22,23]. Recognition of LTB_4_ through its cognate receptor Type 1 (BLT1) enhances antimicrobial effector functions and production of cytokines in phagocytic cells [22,24]. Moreover, LTB_4_ acts as a phagocyte chemoattractant and a trigger of chronic inflammation [24,25]. However, when produced in high amounts, LTB_4_ can be detrimental to host defense [15]. We and others have shown that aberrant LTB_4_ production also exacerbate the inflammatory response, promote insulin resistance and delays skin wound healing [13,14,26].

On the other hand, PG elicits a wide range of biological effects associated with inflammation [23]. The synthesis of PGE_2_ is upregulated and it may signal through four different primary receptors. The concentrations of PGE_2_ and specific receptor signaling are crucial to induce proinflammatory or regulatory immune responses [23]. Several studies showed that PGE_2_ impairs the function of innate phagocytes, rendering the host more susceptible to bacterial, fungal, viral infections and protozoan parasites [20,27–29]. Regarding parasite infections, PGE_2_ has been implicated in the downregulation of ROS production from phagocytes, compromising a protective host response against *Leishmania* [20,30]. In an opposite way, LTB_4_ potentiates phagocytes leishmanicidal activity, through increased secretion of ROS [18,19]. As an intracellular parasite, the control of *Leishmania* infection relies on the balance between LTB_4_ and PGE_2_ production by phagocytes. Thus, unbalanced levels of inflammatory lipid mediators can be detrimental to host response, compromising the elimination of the parasite and influencing disease outcome.

Here, we hypothesize that the low-grade inflammation observed in patients with diabetes increases susceptibility to skin infections and compromises the response to treatment. We assessed CL subjects with diabetes and we found that LTB_4_ correlates with delayed healing time. In addition, macrophages from individuals with diabetes are more susceptible to *L. braziliensis* infection, due to an unbalanced LTB_4_/PGE_2_ production, resulting in inefficient ROS release. Together, our data show that diabetes induces a systemic inflammatory environment causing an inefficient local immune response against *L. braziliensis*, rendering subjects with diabetes more prone to this infection with a worse prognosis.

## Materials and methods

### Ethics statement

The Institutional Board for Ethics in Human Research at the Gonçalo Moniz Institute (Oswaldo Cruz Foundation-IGM-FIOCRUZ, Salvador, Bahia-Brazil), approved this study (protocol number: CAAE 95996618.8.0000.0040). Moreover, all data and samples had the consent of the participants.

### Study area

The present cross-sectional cohort study was conducted in the Corte de Pedra district, an endemic area for cutaneous leishmaniasis caused by *Leishmania braziliensis,* located in the municipality of Tancredo Neves (state of Bahia) in northeastern Brazil. The recruitment was conducted between the years 2015–2018, with biweekly visits of our team of physicians to the endemic area.

### Sample collection

Prior to therapy, blood and skin biopsy samples were obtained from CL subjects with diabetes (CL + DM) or not (CL). For cell culturing and inflammatory mediator quantification, blood samples (20 mL) were drawn by venipuncture using tubes with Heparin, centrifuged for obtaining of the plasma and Peripheral Blood Mononuclear Cells (PBMCs) using HISTOPAQUE® 1077 (Sigma Aldrich, St Louis, MO). For histopathological analysis, skin biopsies were obtained using a 4 mm punch at borders of topical lesions.

### Study population and disease diagnosis

The primary outcome variable was blood glucose levels in individuals with CL. A convenience sample of eight subjects with confirmed CL and 12 individuals with diabetes and CL were enrolled from the Municipal Health Clinic located in Corte de Pedra (CSCP). The statistical power was estimated using Epi infoTM software. This approach was carried out considering a 95% confidence interval (two-sided). The power estimated for each parameter measured in this study was above 80%.

The groups were paired through age and sex. CL diagnosis was performed from clinical analyzes, PCR for parasite DNA detection in lesion biopsies and Montenegro skin test. Fasting glucose levels were used to determine diabetes status: glucose levels ≥126 mg/dL were considered with diabetes (CL + DM), while values under this cutoff were considered without diabetes (CL). Exclusion criteria: pregnant women, children under 15 years old and PCR negative for parasite detection. All samples were collected after the diagnosis of leishmaniasis and before starting the treatment. As recommended by the guidelines of the Brazilian Ministry of Health, the treatment of CL was performed with Glucantime cycles and patients with diabetes were also treated with Metformin. Clinical and epidemiological information was also obtained from all included individuals, e.g. gender, age, lesion size, number and time to healing (see electronic supplementary Table S1).

### Detection of inflammatory mediators

Cytokines TNF-α (Invitrogen, San Diego, CA), IL-1β, IL-6 and IL-10 (all from eBioscience, San Diego, CA) were quantified using sandwich enzyme-linked immunosorbent assays in accordance with the manufacturer’s instructions. To measure LTB_4_ and PGE_2_ levels, competitive assays were used (Leukotriene B_4_ ELISA Kit and Prostaglandin E_2_, both from Cayman Chemical, Ann Arbor, MI), following the manufacturer’s instructions.

### Gene expression in the lesion

The relative expression of Arachidonate 5-Lipoxygenase (*ALOX5*) and Prostaglandin-Endoperoxide Synthase 2 (*PTGS2*) was assessed in biopsies of seven patients with CL and four patients with CL + DM available in RNAlater (Thermo Fisher Scientific, IL, USA), for this approach. The tissues were lysed using TRIzol reagent (Invitrogen, San Diego, USA). Subsequently, phenol–chloroform extraction and quantification of total RNA were performed. cDNA was synthesized by mRNA reverse transcription (RT) using a SuperScript® III Reverse Transcriptase kit (Invitrogen, San Diego, CA, USA). Then, cDNA was amplified by quantitative real time PCR (RT-qPCR) using SYBR Green PCR Master Mix (Thermo Fisher Scientific, IL, USA). The fold change of expression between CL and CL + DM patients was calculated using the 2^-ΔΔCT^ method after normalization with the housekeeping gene β-actin. Specific primers for *ALOX5* (Hs.PT.56a.28007202.g), *PTGS2* (Hs.PT.58.77266) and *ACTB* (Hs.PT.39a.22214847) were purchased from IDT (Illinois, USA).

### Histopathological analysis

Skin biopsies were stained with Hematoxylin and Eosin (H&E) to analyze cell infiltrate and the presence of parasites. Images from five randomly selected fields were captured and scanned using an Olympus Scanner under 40x magnification. Within the software, 50% digital magnification was made to better visualize. The quantification of neutrophils and amastigotes was performed manually using blinded counts, with aid of ImageJ 1.52a software (National Institutes of Health, USA).

### Culturing of human macrophages

PBMCs from CL and CL + DM were plated (2 × 10^6^) on 24-well tissue culture plates (TPP, Trasadingen, Switzerlan) using 13 mm round glass coverslips (Perfecta). Plates were incubated at 37°C under 5% CO_2_ for 30 min. Non-adherent cells were removed by washing and adherent cells were cultured in RPMI 1640 medium (LGC Biotecnologia, São Paulo, Brazil) with 10% autologous serum, 2 mM L-glutamine, 100 U/mL penicillin, 100 mg/mL streptomycin and 50 ng/mL M-CSF for 7 days. Glucose was added to cell cultures at the identical concentrations measured in the blood of each patient.

### Leishmania braziliensis and macrophages infection

*L. braziliensis* (MHOM/BR/01/BA788) promastigotes were cultured at 24°C in Schneider’s Insect medium (Sigma Aldrich, St Louis, MO) supplemented with 10% FBS, 2 mM L-glutamine, 100 U/mL penicillin and 100 mg/mL streptomycin. Cultured macrophages were infected for 4 h with stationary-phase *L. braziliensis* at a parasite/cell ratio of 10:1. The cells were fixed, stained using the Quick Panoptic (LB Laborclin, Paraná, Brazil) and analyzed by light microscopy under an objective (Nikon) at 100x to determine infection rate and the presence of amastigotes by blinded counts.

### Reactive oxygen species production

ROS production was measured following the infection of monocyte-derived macrophages. Cells were incubated for 30 min with 5 μM of CellROX™ Green Reagent (Thermo fisher Scientific⁠, IL, USA) at 37°C under 5% CO_2_. Next, cells were washed three times with 1x PBS (Gibco), fixed and stained with DAPI and ProLong Gold antifade reagent (Thermo fisher Scientific⁠, IL, USA). Images were captured under a fluorescence microscope (Leica DMi8) with 485/520 excitation. Corrected Total Cell Fluorescence (CTCF) was calculated based on an average of 24 cells (eight randomly selected cells per field) for each individual using ImageJ 1.52a software (National Institutes of Health, USA).

### Statistical analysis

The Mann–Whitney test was used to compare groups with unpaired samples, such as systemic inflammatory mediators (LTB_4_, PGE_2_, TNF-α, IL-6, IL-1β and IL-10), histological analysis, gene expression and infection rate. Kruskal–Wallis with Dunn’s post-test was used to compare more than two groups (non-infected, infected, CL and CL + DM). For comparisons involving paired samples, such as ROS production, LTB_4_ and PGE_2_ from culture supernatant, Wilcoxon’s test was used. Trend analysis was performed using the Chi-squared test for amastigotes number in the lesion. Correlation analysis was employed considering clinical parameters, such as blood glucose, lesion size, healing time and inflammatory mediator levels, obtained from Spearman’s rank test. One individual with missing data (healing time) is shown in electronic supplementary Table S1. All statistical analyses were performed using GraphPad Prism 7.0 (GraphPad, San Diego, CA). Differences were considered statistically significant when *p* < 0.05.

## Results

### CL + DM individuals present increased systemic levels of inflammatory mediators

**Figure 1.**
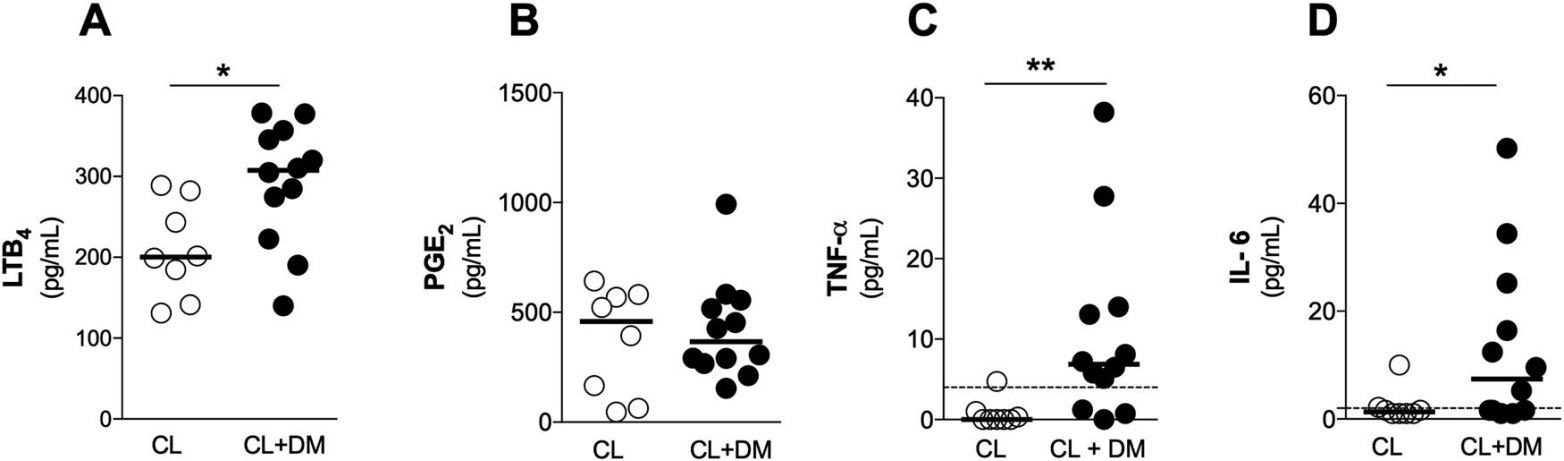
DM increases systemic levels of inflammatory mediators in CL individuals. (A) LTB4, (B) PGE_2_, (C) TNF-*a* and (D) IL-6 levels in plasma of individuals with Cutaneous Leishmaniasis, without diabetes (CL) or with diabetes (CL + DM) measured by ELISA. CL, n = 8; CL + DM, n = 12. Data shown in median. Mann-Whitney test. *p < 0.05; **p < 0.01.

People with diabetes exhibit increased systemic inflammatory cytokines that might be involved in different comorbidities [2,12]. Initially, we obtained plasma samples from individuals with CL and CL + DM to measure systemic levels of inflammatory mediators. CL + DM individuals presented elevated plasma levels of LTB_4_ (Figure 1(A)), TNF-*a* (Figure 1(C)) and IL-6 (Figure 1(D)) when compared to CL. However, no differences were found in PGE_2_ (Figure 1(B)), IL-1β and IL-10 (levels below the detection limit, data not shown) between CL + DM and CL individuals. These findings suggest that diabetes induces an exacerbated and systemic inflammatory profile during CL.

### Blood glucose positively correlates with inflammatory mediators, worsening the healing time in CL + DM

**Figure 2.**
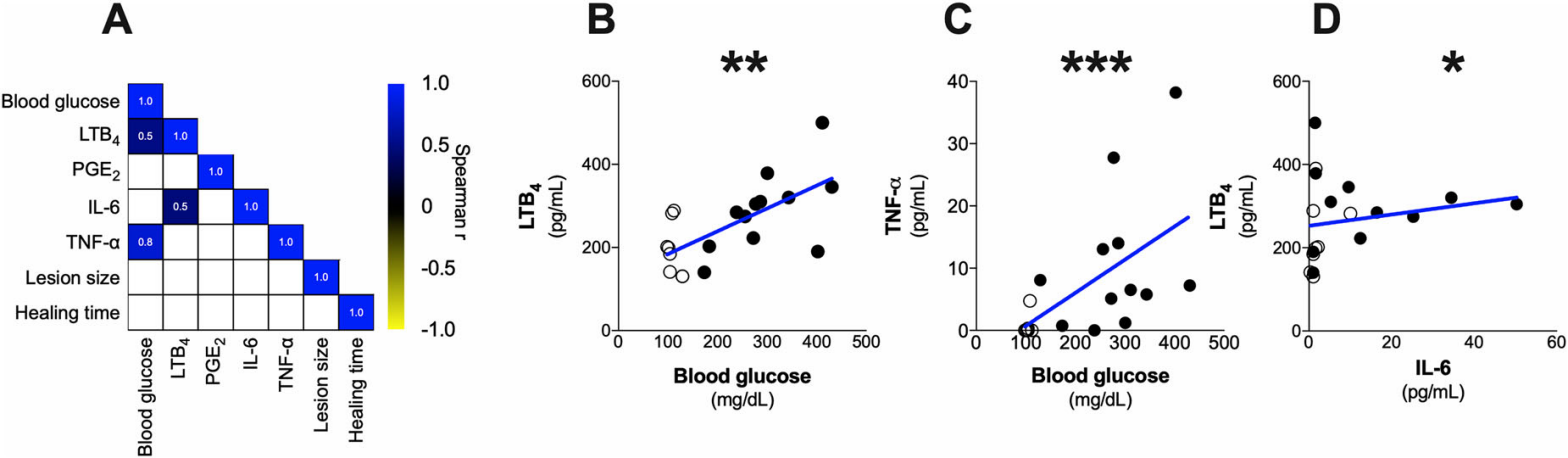
Blood glucose positively correlates with inflammatory mediators in CL individuals regardless of diabetes. (A) Correlation matrix of the data obtained from CL (open circles) and CL + DM (full circles) individuals grouped together. Detailed correlation between blood glucose and LTB_4_ (B), TNF-*a* (C) and between LTB_4_ and IL-6 (D). Spearman correlation. *p < 0.05; **p < 0.01; ***p < 0.001.

Next, we determined whether the abundance of inflammatory mediators correlate with clinical aspects, such as lesion size, wound healing time as well as blood glucose levels in all patients grouped together (both CL and CL + DM). Despite clinical parameters are statistically similar between CL and CL + DM groups (except plasma glucose levels – see electronic supplementary Table S1), the role of inflammatory mediators could be specific for each condition (with or without diabetes). The matrix summarizes the statistically significant correlations found between these parameters (Figure 2(A)). As shown in Figure 2, the graphs detail all significant correlation found between the parameters, such as blood glucose levels and LTB_4_ (*r* = 0.5) (Figure 2(B)), as well as TNF-α (*r* = 0.8) (Figure 2(C)) regardless of diabetes status in subjects with CL. Furthermore, our results showed that LTB_4_ production was positively correlated with IL-6 (*r* = 0.5) (Figure 2(D)). Therefore, these results indicate that glycemic levels and serum LTB_4_ could contribute to systemic inflammation.

**Figure 3.**
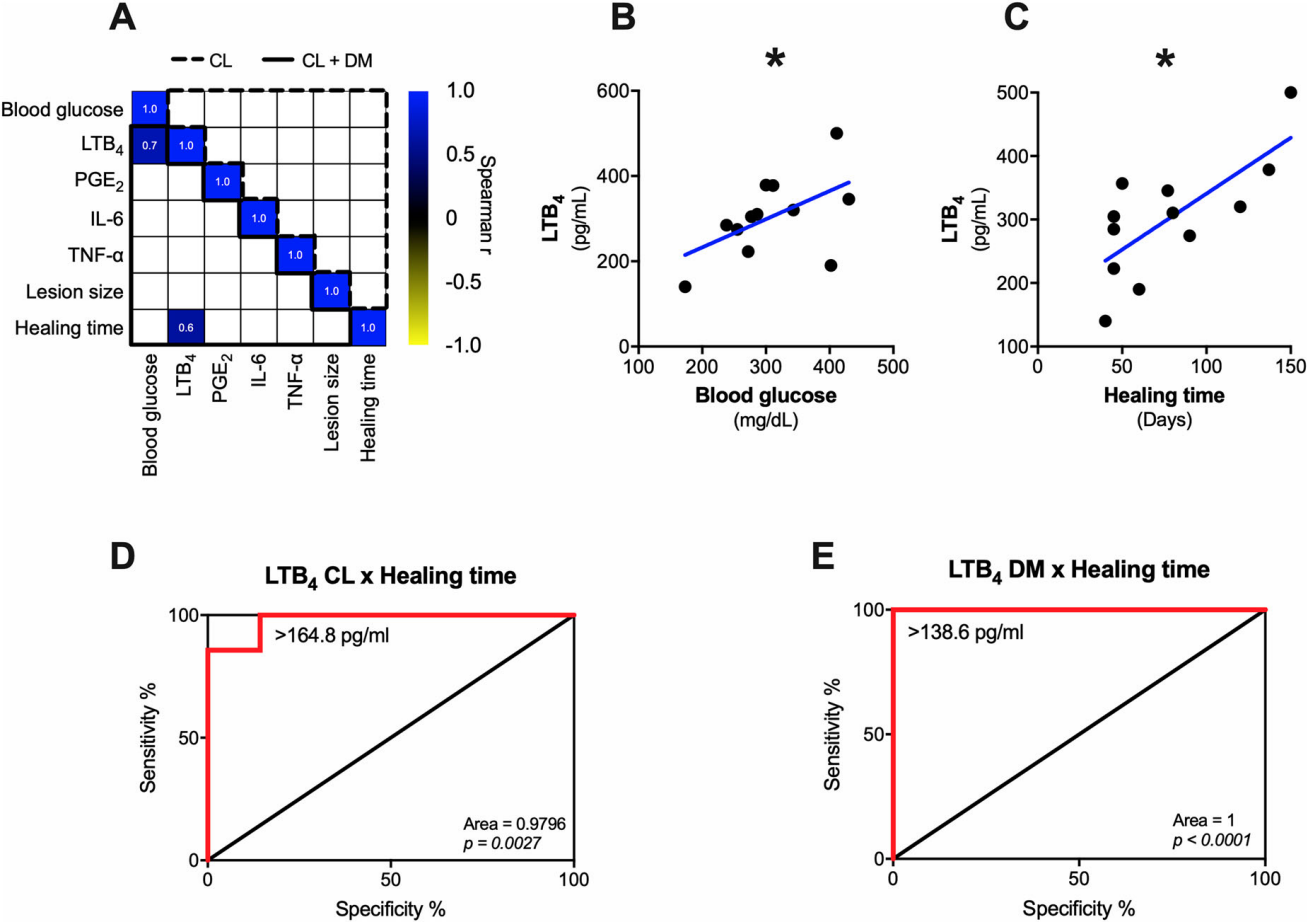
Only LTB_4_ positively correlates with healing time and blood glucose in CL + DM individuals. (A) Correlation matrix of clinical data and inflammatory mediators in CL (dashed line) and CL + DM (full line) individuals separately. Detailed correlation between LTB_4_ and blood glucose (B) and healing time (C) in CL + DM patients. Receiver operating characteristic (ROC) curve of LTB_4_ participation in healing time of (D) CL and (E) CL + DM individuals. Spearman correlation (A-C). *p < 0.05.

To understand the influence of diabetes on CL, we sought for correlations in CL and CL + DM groups separately (Figure. 3(A)). As shown in Figure 3(A), no correlation was observed in CL subjects (dashed line). However, only CL + DM individuals (full line) show a positive correlation of LTB_4_ production with blood glucose (*r* = 0.7) (Figure 3(B)) and healing time (*r* = 0.6) (Figure 3(C)). We further confirmed that these correlations are not present in CL individuals (see electronic supplementary Figure S1). These results suggest that hyperglycemia-induced LTB_4_ could drive the delayed healing time in CL + DM individuals, but other mediators might also play a role.

We further performed a Receiver Operating Characteristic curve (ROC) analysis to evaluate the role of systemic LTB_4_ levels in the lesion outcome (healing time) between CL (Figure 3(D)) and CL + DM (Figure 3(E)) individuals. The results showed that LTB_4_ systemic levels above 164.8 pg/mL (specificity 100% and sensitivity 85.71%) influence the healing time of CL subjects (Figure 3(D)). On the other hand, for CL + DM individuals, these levels were above 138.6 pg/mL (specificity and sensitivity 100%) (Figure 3(E)).

These findings suggest that the threshold of systemic LTB_4_ levels in CL + DM individuals is lower than CL subjects to delay the healing time. We can hypothesize that individuals with diabetes are more responsive to LTB_4_ effects, but further studies with prospective cohorts are necessary to confirm these results.

### Lesions from CL + DM individuals present a neutrophilic infiltrate and reduced 5-LO expression

**Figure 4.**
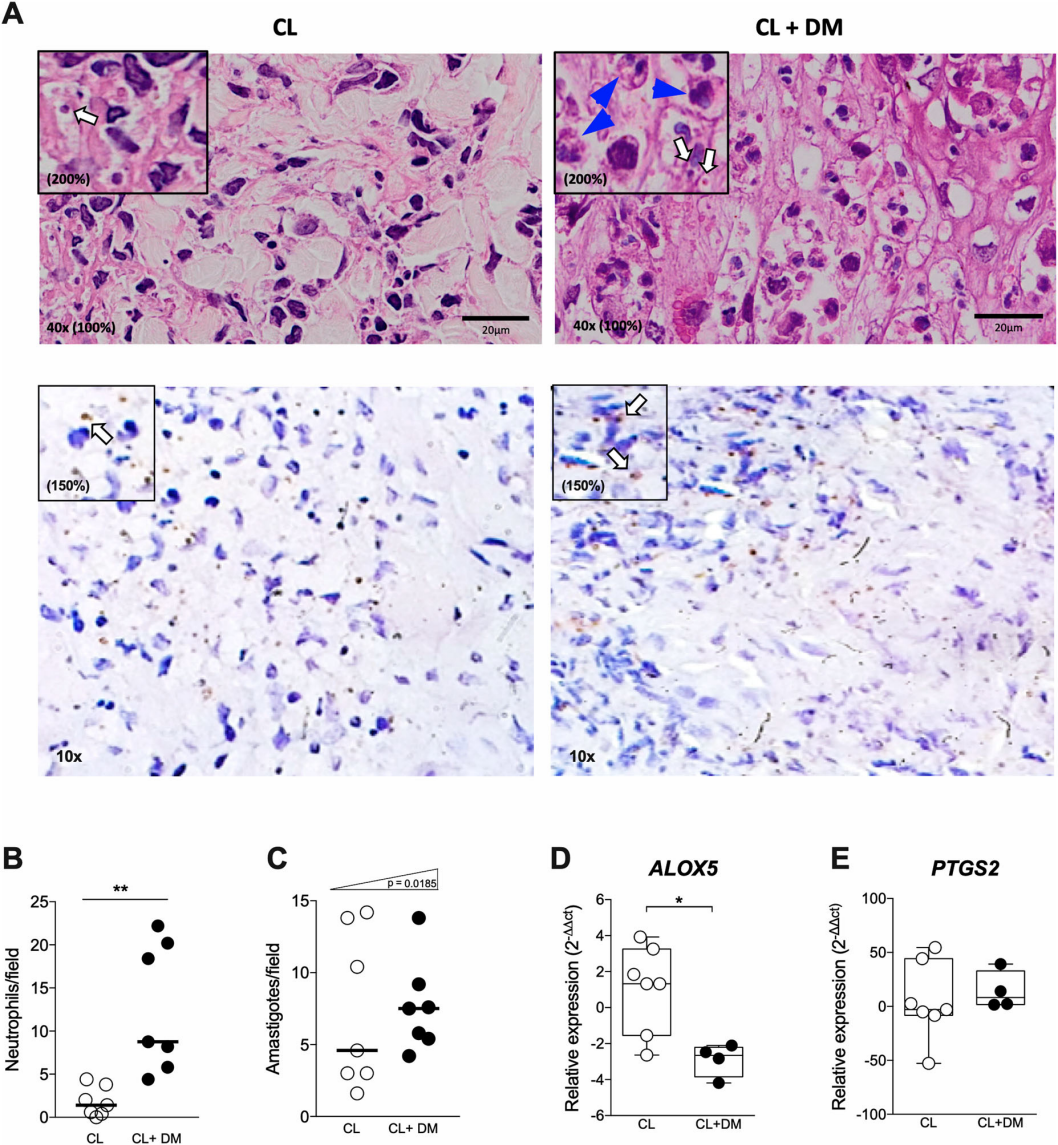
DM individuals are more prone to CL infection. (A) Histological images of a representative CL (left) and CL + DM (right) lesions in HE (above) and IHQ (below). Quantification of neutrophils (B) and amastigotes for field (C) in the lesions of CL and CL + DM individuals. Expression of *ALOX5* (D) and *PTGS2* (E) in the lesion of CL and CL + DM individuals detected by RT-qPCR. Blue arrows: neutrophils; white arrows: amastigotes. Scale bar: 20 µm. 40x objective with 200% digital magnification in HE and 150% in IHQ. Data shown in median (B,C). Box and whiskers with min to max (D,E). Mann-Whitney test (B,D). Chi-squared test for trend (C). *p < 0.05; **p < 0.01.

Next, we sought to locally identify the inflammatory profile in CL and CL + DM lesions, since the production of lipid mediators and the cellular infiltrate could define susceptibility to *Leishmania* spp*.* [20,31]. Representative images of CL lesions (Figure 4(A)) show the cellular infiltrate of CL subjects (left) and CL + DM individuals (right). We observed a significant increase of neutrophil infiltrate in CL + DM compared with CL lesions (Figure 4(B)). Furthermore, we found a trend toward higher numbers of amastigotes per field in CL + DM lesions (Figure 4(C)). The gene expression of enzymes that produce inflammatory lipid mediators, such as LTB_4_ (*ALOX5* encodes 5-LO enzyme) and PGE_2_ (*PTGS2* encodes COX-2 enzyme), was assessed in these lesions. Surprisingly, the results revealed a reduction in the expression of *ALOX5* in CL + DM compared to CL (Figure 4(D)). On the other hand, *PTGS2* gene expression was not altered in patients with diabetes (Figure 4(E)). Together, these findings suggest that diabetes locally alters the inflammatory infiltrate with a reduction of 5-LO expression in the lesion and increased susceptibility to CL.

### Macrophages from CL + DM individuals are more susceptible to L. braziliensis infection

**Figure 5.**
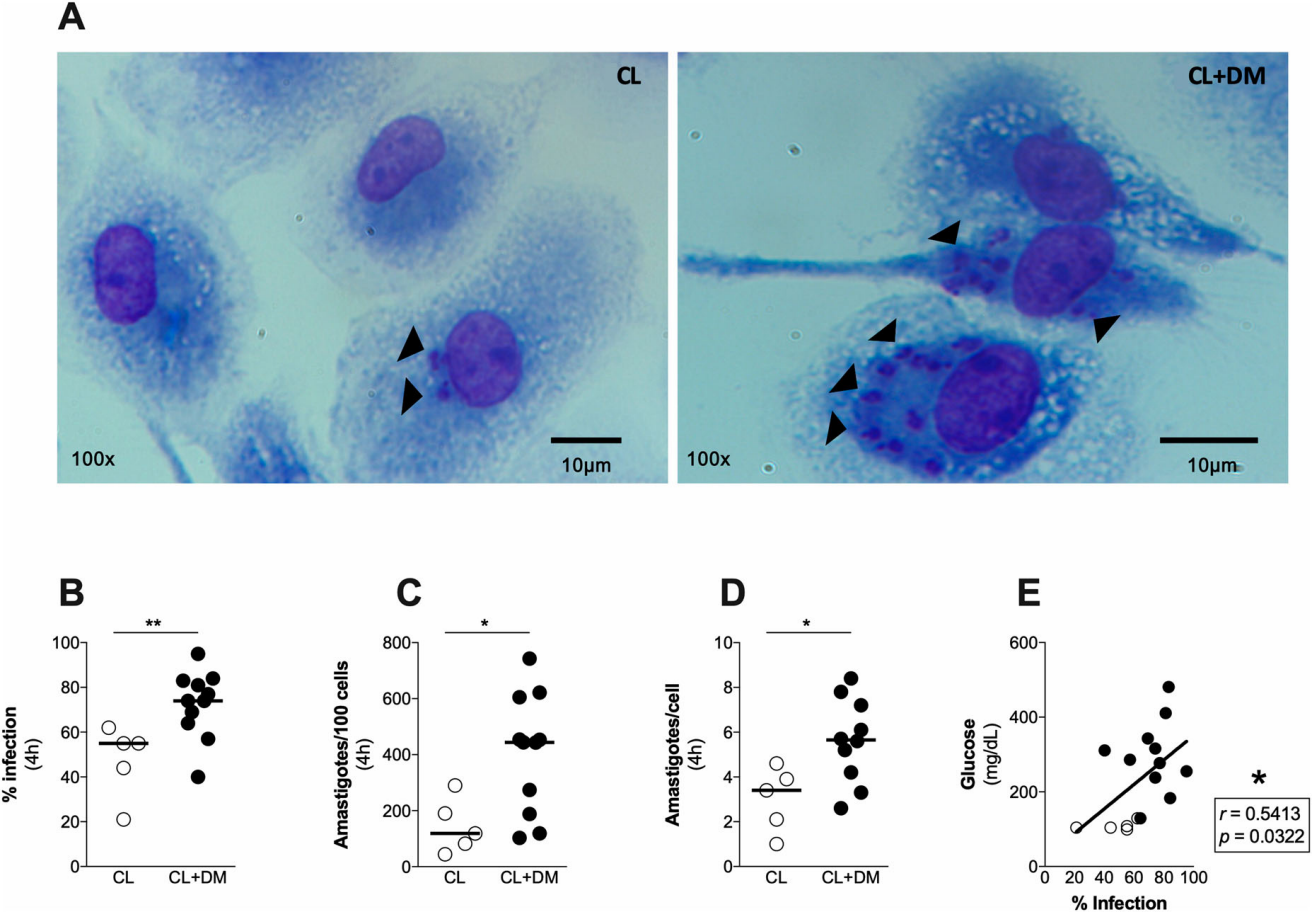
Monocyte-derived macrophages from individuals with CL + DM are more susceptible to *Leishmania braziliensis*. Representative images of cultured macrophages from CL and CL + DM individuals after *L. braziliensis* infection (A). Quantification of infection rate (B), amastigotes in 100 cells (C) and amastigotes per cell (D) in macrophages from CL and CL + DM individuals, after infection with *L. braziliensis*. (E) Spearman correlation between blood glucose levels and infection rate in the CL (open circles) and CL + DM (full circles). Arrows indicate the presence of amastigotes. Data shown in median. Mann-Whitney test (B-D). Spearman correlation (E). *p < 0.05; **p < 0.01.

Since we found an increased susceptibility to *L. braziliensis* in the lesions of CL + DM, we assessed the response of macrophages from these individuals *in vitro* (Figure 5(A)). It is well-established that *Leishmania* parasites reside in macrophages, which control the infection through the production of reactive oxygen species (ROS), cytokines and chemokines [18]. We found an increased infection rate (Figure 5(B)), total number of intracellular amastigotes (Figure 5(C)) and amastigotes per macrophage (Figure 5(D)) in CL + DM compared to CL macrophages. Furthermore, a positive correlation was observed between blood glucose levels and infection rate of macrophages (Figure 5(E)), suggesting that hyperglycemia leads to susceptibility to *L. braziliensis* in macrophages.

### ROS production is unaltered by L. braziliensis infection in CL + DM macrophages

**Figure 6.**
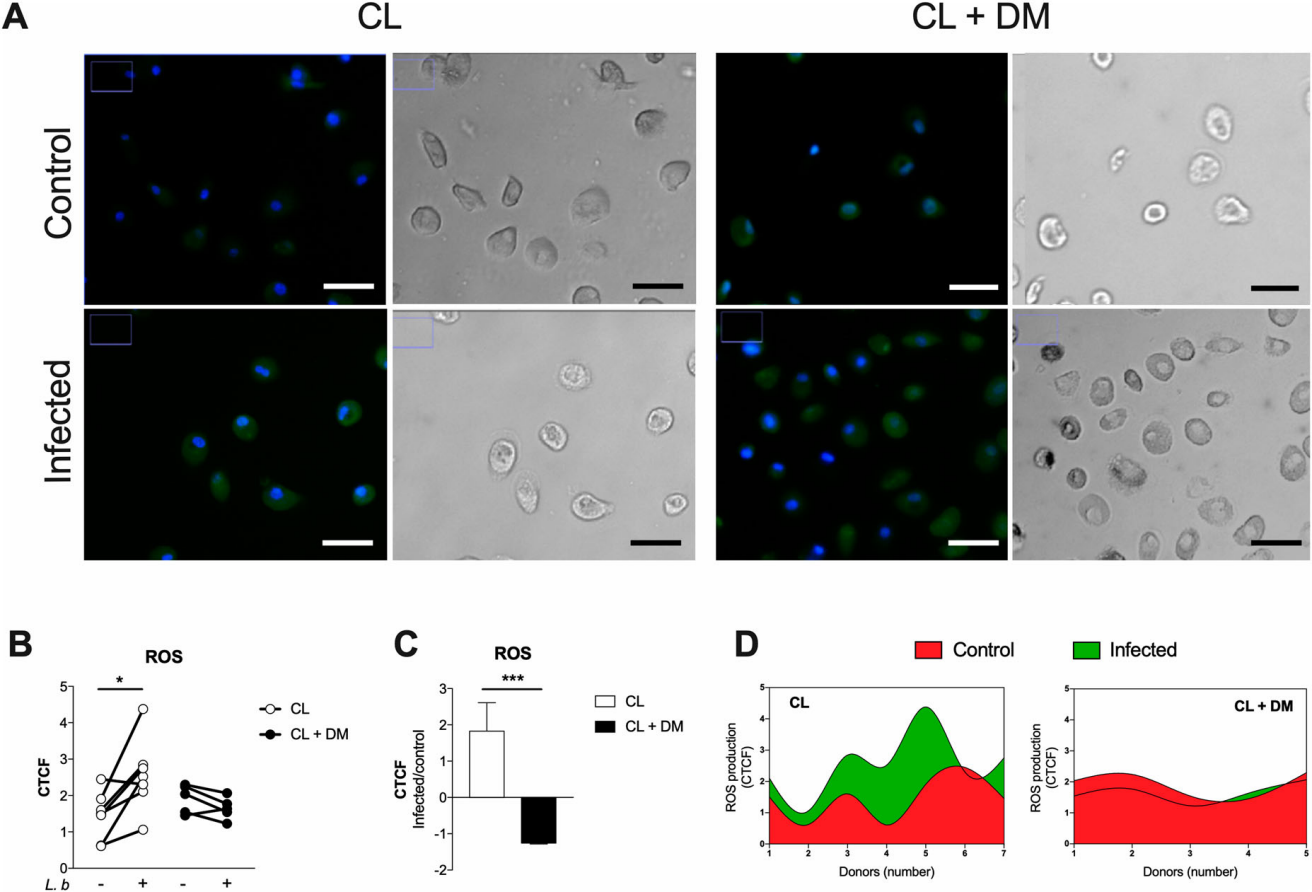
Unaltered production of ROS after *L. braziliensis* infection of monocyte-derived macrophages from CL + DM individuals*.* Representative images of ROS production in monocyte-derived macrophages from CL and CL + DM individuals, before (control) and after infection by *L. braziliensis* (infected) (A). Quantification of ROS production in monocyte-derived macrophages from CL (open circles) and CL + DM (full circles) individuals after *L. braziliensis* infection (B). Ratio of ROS production between infected and control samples of CL and CL + DM individuals (C). Cubic spline analysis of donor-specific ROS production in monocyte-derived macrophages before (red) and after (green) *L. braziliensis* infection from CL and CL + DM individuals. CTCF: Corrected Total Cell Fluorescence. Green: ROS production; blue: cell nucleus. 40x magnification. Scale bar = 10 µm. Data represent individual values before and after infection (B). Data shown in median with interquartile range (C). Wilcoxon’s test (B). Mann-Whitney test (C). *p < 0.05; ***p < 0.001.

To investigate the mechanisms underlying the increased susceptibility of CL + DM macrophages to *L. braziliensis*, we measured the production of ROS. Representative images of cultured macrophages before and after *L. braziliensis* infection show ROS labelling in green (Figure 6(A)) comparing CL (left) and CL + DM (right). We found that ROS production of macrophages from CL + DM individuals (dark circles) is unaltered after *L. braziliensis* infection (Figure 6(B)). On the other hand, macrophages from CL (clear circles) subjects efficiently respond to the infection with increased ROS production. Moreover, the ratio of ROS production between infected and control macrophages revealed higher ROS production in CL subjects (Figure 6(C)). The production of ROS is also showed individually through Cubic spline analysis (Figure 6(D)), confirming that CL + DM macrophages do not alter its production after *L. braziliensis* infection. Taken together, these findings suggest that, under diabetes, macrophages exhibit a reduced capacity to produce ROS in response to *L. braziliensis* infection.

### CL + DM macrophages produce more PGE_2_ than LTB_4_ in response to L. braziliensis infection

**Figure 7.**
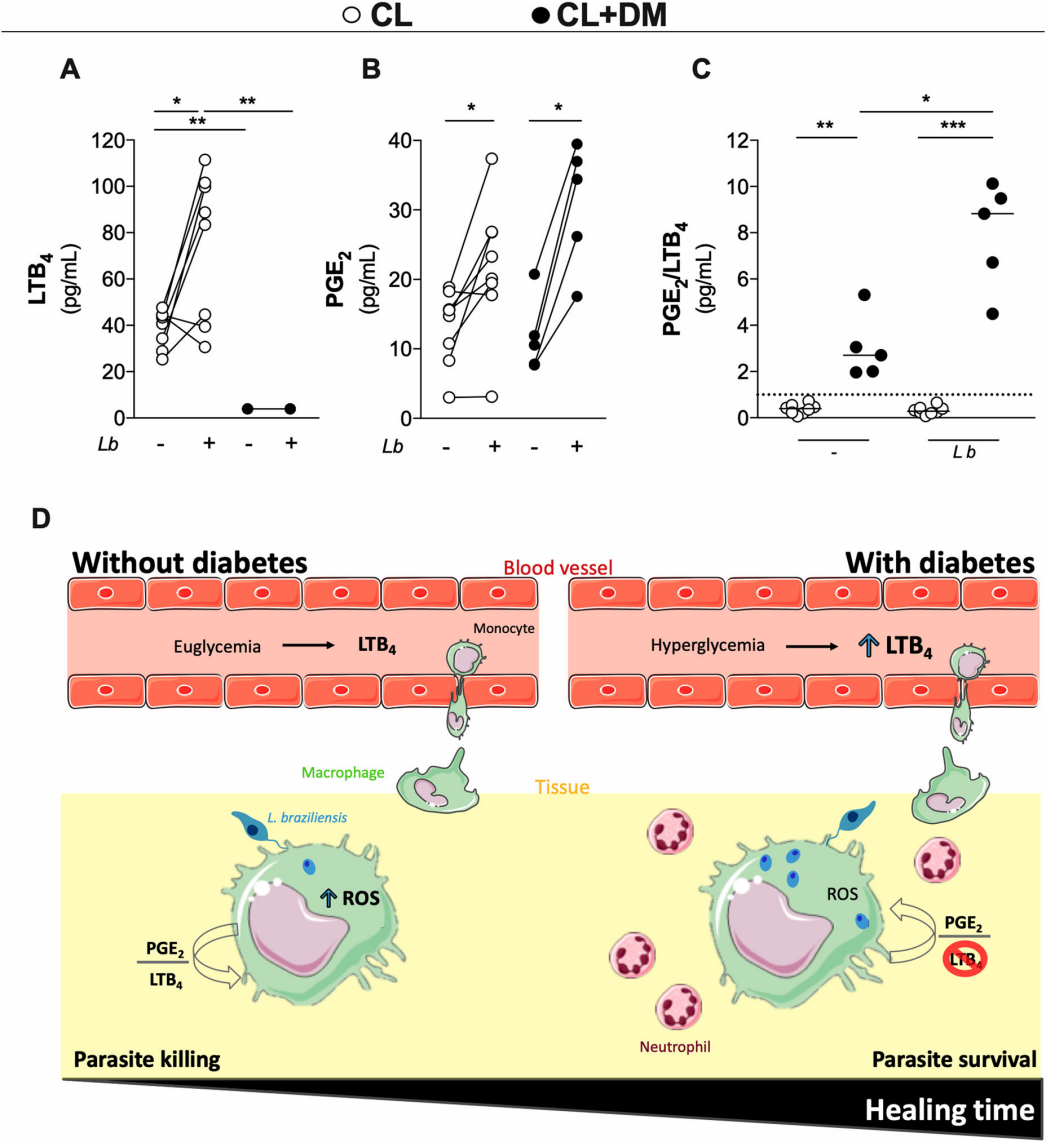
LTB_4_ production of monocyte-derived macrophages from CL + DM individuals is abolished after *L. braziliensis* infection. Production of LTB_4_ (A) and PGE_2_ (B) in the supernatant of cultured macrophages from CL and CL + DM individuals after infection with *L. braziliensis.* Ratio between PGE_2_ and LTB_4_ production in the supernatant of uninfected or infected macrophages (C). Graphical abstract (D). Data represent individual values before and after infection. Wilcoxon’s test and Kruskal-Wallis with Dunn’s post-test. *p < 0.05; **p < 0.01.

To investigate whether ROS release could be a consequence of lipid mediators production, we measured LTB_4_ and PGE_2_ in the culture of macrophages from CL and CL + DM individuals. It is well established that the balance between LTB_4_ and PGE_2_ can influence the outcome of *Leishmania* spp*.* infection [32]. Our findings revealed negligible levels of LTB_4_ in CL + DM macrophages in response to *L. braziliensis* infection, while levels of LTB_4_ in CL macrophages increased after infection (Figure 7(A)). By contrast, both CL + DM and CL macrophages produced increased levels of PGE_2_ after infection (Figure 7(B)). To define the predominance of these mediators in the microenvironment, the ratio between PGE_2_ and LTB_4_ was calculated in both conditions, before and after infection. Interestingly, PGE_2_ was the more predominant lipid mediator in CL + DM macrophages, regardless of infection status (Figure 7(C)). Together, these findings show that diabetes imbalances PGE_2_/LTB_4_ ratio locally produced from macrophages after *L. braziliensis* infection probably through a negative feedback from increased systemic inflammation.

In sum, this study assessed systemically and locally the effects of diabetes in CL outcome. We found basal levels of LTB_4_ in the bloodstream of CL subjects and increased LTB_4_ over PGE_2_ production from macrophages after *L. braziliensis* infection, resulting in ROS release of and parasite killing. On the other hand, CL + DM individuals show increased levels of LTB_4_ in the bloodstream. Furthermore, diabetes abolished LTB_4_ production from their macrophages driving PGE_2_/LTB_4_ ratio toward PGE_2_ predominance. This imbalance prevents the production of ROS, favouring parasite survival, increasing the number of neutrophils in the lesion and delays CL healing time (Figure 7(D)).

## Discussion

In the current study, we show that diabetes induces a systemic inflammatory profile in individuals with CL. We found that glycemic status and LTB_4_ levels directly affect the healing time of CL lesions. More importantly, macrophages obtained from CL + DM individuals are more susceptible to *L. braziliensis* infection.

Herein, we assessed circulating levels of inflammatory mediators in the blood of CL and CL + DM individuals. We found an increase in TNF-α, IL-6 and LTB_4_ systemic levels of CL + DM. It has been shown that LTB_4_ is produced immediately in response to infections, and is one of those responsible for the production of proinflammatory cytokines in experimental model of diabetes [33–35]. Given that inflammatory lipid mediators, such as LTB_4_ and PGE_2_, have been scarcely studied in human with diabetes, these findings represent an initial result of diabetes-induced LTB_4_ production in response to a protozoan parasite in humans. Interestingly, although our data show an increased production of different inflammatory mediators in CL + DM individuals, only LTB_4_ positively correlates with blood glucose levels and healing time of CL lesion. Similar results were described in an experimental model of diabetes, where LTB_4_ was found to promote insulin resistance [33]. Accordingly, mice deficient for 5-lipoxygenase ⁠(5-LO), presented faster healing time of a sterile skin wound compared to wild type mice, indicating that LTB_4_ may be an important target to improve cutaneous healing [36]. Moreover, a previous study demonstrated that exaggerated LTB_4_ signaling correlated with larger nonhealing lesion areas and increased bacterial loads in diabetic mice infected with ⁠methicillin-resistant *Staphylococcus aureus* (MRSA) in the skin [15]. We therefore expanded these findings to humans, confirming that diabetes induces a systemic inflammatory profile and delayed healing of a skin lesion caused by an infectious agent.

The healing of an injured tissue is a complex process, involving several inflammatory mediators and immune cells, such as neutrophils, the first leukocytes recruited, followed by monocytes and lymphocytes [15,36–38]. Enhanced neutrophil chemotaxis is dependent on complement fragments, chemokines and exogenous mediators produced by pathogens, as well as the presence of LTB_4_. More specifically, LTB_4_ signaling can increase ⁠actin polymerization and myosin phosphorylation, which are crucial to neutrophil migration [25,38]. Our findings indicate that diabetes induces an increased neutrophil infiltrate to the skin lesion caused by *L. braziliensis*. Similarly, high levels of LTB_4_ were associated with a predominant neutrophil infiltrate toward a sterile injury in the skin of diabetic mice [34]. Regarding skin lesions caused by an infectious agent, it was observed an inefficient abscess formation against *S. aureus* in an experimental model of diabetes, where LTB_4_ was associated with altered neutrophil migration [15]. Taken together, these findings indicate that LTB_4_ orchestrates an exaggerated systemic inflammatory response with neutrophil predominance in the infiltrate, which could be related with the delayed healing process observed in skin lesions of individuals with diabetes.

Diabetes is also known to promote susceptibility to infections by extracellular bacteria, fungus and mycobacteria [39–41]. However, the susceptibility to a parasitic infection remains poorly understood. Indeed, we found a trend towards higher numbers of amastigotes in the lesions of CL + DM individuals. With an increment in the number of lesion samples, we should detect statistically differences between the groups. In addition to the small number of patients included, our study is also limited by the lack of a molecular tool to quantify parasite burden in lesions in order to confirm increased susceptibility in CL + DM individuals. However, direct blinded counts of amastigotes in lesions stained with HE represents a reliable alternative approach. Further investigation will be necessary with increased sample sizes to quantify parasite load by molecular methods to confirm whether diabetes favours *L. braziliensis* survival.

In accordance with these findings in the lesion, locally expression of *ALOX* gene, which encodes 5-LO enzyme [22], is reduced in CL + DM individuals, compared to CL samples, indicating a local down-modulation of LTB_4_ mediated response. It is important to consider that diabetes involves chronic low-grade inflammation, triggered by endogenous molecules [42]. The accumulation of metabolic products, such as glucose, can induce “sterile inflammation” through the engagement of TLRs [43] or inflammasome activation [44]. Despite the inflamed environment, this immunological dysfunction renders individuals with diabetes more prone toward infectious diseases [40]. Since the main pathogenic mechanism of increased susceptibility in diabetes is a hyperglycemic/inflamed systemic environment that reduces the production of mediators locally in response to infection, this implies the reduction of chemotaxis, phagocytic activity and immobilization of polymorphonuclear leukocytes [40].

We speculate that LTB_4_ may be provoking a counterintuitive effect in the context of diabetes, i.e. the excessive production of this mediator is associated with a deficient phagocyte response to pathogens [13,15]. While the mechanisms involved in this association remain unclear, it is theoretically possible that chronic exposition to hyperglycemia could lead to alterations in epigenetic marks or to trained immunity, causing macrophages to respond to subsequent restimulation in a sensitized and non-specific manner [45].

During inflammation, the production of leukotrienes is increased, as already reported for diabetic mice, that exhibited high serum levels of LTB_4_ [46]. However, the source of this increased systemic levels of LTB_4_ is a matter of debate. The production of leukotrienes is largely confined to leukocytes, mainly neutrophils and monocytes/macrophages [22], but other cell types can take up products from arachidonic acid and metabolize it into bioactive leukotrienes through transcellular biosynthesis, such as endothelial cells and keratinocytes [26]. Considering that diabetes and CL are chronic diseases, these individuals probably produce LTB_4_ in a constitutive manner [47]. Although we and others have studied LTB_4_ production by macrophages [13], other cell types could be involved, such as neutrophils. The CL lesion is initially composed of neutrophils that are later replaced by an intense macrophage infiltrate. Nevertheless, the source of LTB_4_ in diabetes and CL remains to be determined.

The main mechanism described to resolve *Leishmania* infection is through the production of ROS by different cell types, especially macrophages [18,20,32]. Surprisingly, we did not find increased ROS production by macrophages from CL + DM individuals after *in vitro* infection, despite their systemic inflammatory status. On the other hand, macrophages from CL subjects showed higher levels of ROS in response to the infection, resulting in parasite killing. Similarly, CL lesions have increased ROS production compared to healthy control skin [48]. Our results indicate that ROS production by macrophages is compromised during diabetes, rendering these individuals more susceptible to a parasite infection. However, phagocytes from diabetic conditions were shown to be able produce increased levels of ROS compared to non-diabetics [13], it was also reported that cells from diabetic patients with tuberculosis produce reduced levels of ROS [49]. Similarly, we showed that, after *L. braziliensis* infection, macrophages from CL + DM patients did not exhibit increased ROS production compared to cells from CL patients, yet basal levels (before infection) were similar. Our findings suggest that locally absent LTB_4_ production by macrophages is reflective of reduced ROS production by these cells, since the LTB_4_/PGE_2_ balance is known to modulate ROS production [18–20].

Several studies have also demonstrated that ROS-dependent elimination of *Leishmania* spp. can be mediated by the lipid mediators LTB_4_ and PGE_2_ [18–20]. Accordingly, we found that macrophages from CL + DM individuals produce negligible levels of LTB_4_ after *L. braziliensis* infection compared to CL macrophages. Moreover, PGE_2_/LTB_4_ ratio revealed the predominance of PGE_2_ in CL + DM macrophages both before and after the infection. A similar study described that the balance between these inflammatory mediators dictates the state of susceptibility or resistance to different species of *Leishmania* [32]. In addition, it has already been demonstrated that increased levels of PGE_2_ produced by macrophages in comparison to LTB_4_ impairs ROS production and *L. infantum* killing [20]. This has also been observed in autoimmune disease, such as rheumatoid arthritis [50] and atherosclerosis [51], even in the absence of an infective pathogen. In the context of diabetes, we suggest that systemic chronic low-grade inflammation creates a localized imbalance in eicosanoid production geared towards a predominance of PGE_2_, which leads to unaltered ROS production and the survival of *L. braziliensis* parasites. Taken together, these results suggest that PGE_2_/LTB_4_ balance dictates the production of ROS and, consequently, the control of an infection.

In summary, the current study revealed that diabetes induces a systemic pro-inflammatory profile in individuals with CL, orchestrated by LTB_4_, which correlates with impaired healing process and ability to eliminate parasites. These findings suggest that LTB_4_ play a central role in the innate immune response against pathogens during diabetes. In particular, due to its capacity to induce ROS production, LTB_4_ has significant influence in the disease outcome, defining a host as resistant or susceptible to *Leishmania*. Moreover, this point out LTB_4_ as a potential target for future interventions designed to minimize the impact of leishmaniasis in the context of human with diabetes.
